# Identifying wildlife corridors for the restoration of regional habitat connectivity: A multispecies approach and comparison of resistance surfaces

**DOI:** 10.1371/journal.pone.0206071

**Published:** 2018-11-07

**Authors:** Canran Liu, Graeme Newell, Matt White, Andrew F. Bennett

**Affiliations:** 1 Arthur Rylah Institute for Environmental Research, Department of Environment, Land, Water and Planning, Heidelberg, Victoria, Australia; 2 School of Life and Environmental Sciences, Deakin University, Burwood, Victoria, Australia; 3 Department of Ecology, Environment and Evolution, La Trobe University, Bundoora,Victoria, Australia; Sichuan University, CHINA

## Abstract

Many large-scale connectivity initiatives have been proposed around the world with the aim of maintaining or restoring connectivity to offset the impacts on biodiversity of habitat loss and fragmentation. Frequently, these are based on the requirements of a single focal species of concern, but there is growing attention to identifying connectivity requirements for multi-species assemblages. A number of methods for modelling connectivity have been developed; likewise, different approaches have been used to construct resistance surfaces, the basic input data for connectivity analyses. In this study we modelled connectivity for a multi-species group of vertebrates representative of heavily fragmented forests in north-central Victoria, Australia. For each species, we used least-cost modelling and compared two alternate resistance surfaces, based on species distribution models and on expert opinion, respectively. We integrated the connectivity results across individual species to obtain a multi-species connectivity map for the region. A resistance surface based on expert assessment of the relative use of land-cover classes by the target species was more informative than one based on species distribution models. The former resulted in pathways more strongly aligned with existing patches and strips of native vegetation. In this region, pathways aligned with streams and their associated riparian vegetation have relatively high ecological potential and feasibility to contribute to regional connectivity for the assemblage of forest vertebrates.

## Introduction

Ecological connectivity provides the capacity for the movements of organisms, for gene flow, and for range shifts [1–4], and thereby is a key factor in the long-term viability of populations, particularly for animal species [5]. In human-dominated landscapes, loss and altered configuration of habitats have substantially modified and decreased connectivity [6], and the resulting isolation limits the ability of populations to respond adequately to perturbations. Measures to address declines in ecological connectivity have received increasing attention as researchers attempt to predict the response of species to climate change and other disturbances [4, 7, 8]. There is broad recognition that the maintenance and restoration of connectivity at landscape scales is crucial for biodiversity conservation [3, 9, 10].

An ideal method for designing connectivity networks for a region would be to identify linkages or corridors based on empirical observations of the movements of particular focal species of concern [11]. However, empirical data of this nature are sparse or not available for most species, and consequently connectivity analyses must rely on models [8], coupled with human judgement. Various forms of models have been introduced for connectivity analyses. The most popular method used to inform the design of habitat linkages is least-cost path modelling [8, 12, 13]. The least-cost path is a contiguous vector of cells that has the lowest cumulative ‘cost’ to an organism, as the path crosses from one point (e.g. a known population or habitat patch) to another [6]. This method has been used in many conservation projects [14–17].

Another popular approach derives from circuit theory [18], which treats the landscape as a two-dimensional surface that supports various levels of conductivity [6]. Circuit theory has been used in numerous studies, especially in relation to landscape genetics [19, 20]. These two approaches (least-cost path and circuit theory) represent different assumptions regarding animal behaviour and connectivity. The former assumes that animals have perfect or near-perfect knowledge of the landscape and choose the optimal path, while the latter considers that movement through a landscape occurs in the form of a random walk. Other methods include those based on graph theory [21, 22], resistant kernel models [23], and individual-based movement models [12].

The approaches described above depend on having sound information on landscape resistance, a measure of the cost of movement of an animal through a location in the landscape [24]. Resistance surfaces can be developed from empirical data on gene flow, genetic distances, habitat use and movement paths [6]; or on the basis of expert opinion [12]. The former typically assume that animals make movement decisions based on the same preferences they use in selecting habitat [24]. However, animal movements may be driven by factors other than resource selection, and this approach can only be considered as a proxy of resistance. A concern with resistance derived from expert opinion is that it is difficult to objectively evaluate performance without empirical information [5, 24]. Analyses that compare the utility of these approaches for constructing resistance surfaces, are required.

Many connectivity studies have focused on a single focal species [1, 25], typically large charismatic mammals, or on groups of closely related taxa [26]. Corridors have been identified for ‘flagship species’ (i.e. high profile species that will attract public support), such as the giant panda (*Ailuropoda melanoleuca*) [27, 28]. Other species have been selected to serve as ‘umbrella species’: if landscapes are sufficiently connected for umbrella species, they should also provide habitat connectivity for many other species [26, 29, 30]. Umbrella species may include those that require connectivity to maintain migratory pathways, such as elephant (*Loxodonta africana*) [31, 32]; or large, wide-ranging species that require substantial areas of habitat to maintain viable populations, such as mountain lion (*Felis concolor*) [33, 34], gray wolf (*Canis lupus*) [35, 36], and grizzly bear (*Ursus arctos*) [34, 37, 38].

More recently, htere -has been growing interest in modelling connectivity for multiple species [39–43]. A reliance on corridors designed for a single species may not meet the differing ecological requirements of coexisting species and ecological processes across the landscape [25, 41, 44], and will be less cost-effective than linkages optimised to benefit multiple species [42]. A range of approaches are being developed for multi-species connectivity. One approach is to merge the resistance surfaces for individual species and identify linkages based on the merged resistance surface [16, 41]. Another is to identify corridors for each species separately and overlay corridor maps to identify locations important to multiple species [43–46]. Alternatively, other studies model the ‘naturalness’ (or landscape integrity–level of human modification) of land cover categories [9, 40, 46] on the assumption that the greater the integrity of the landscape the more permeable it will be for multiple species.

In Australia many connectivity initiatives have been proposed [47–49], including large ‘continental-scale’ (i.e. >700 km) corridors [50]. The proposed Great Eastern Ranges (GER) corridor, for example, is 3600 km in length from western Victoria through New South Wales and the Australian Capital Territory to far north Queensland.

The aim of this study was to develop an approach for producing a multi-species connectivity map for a selected region in north-central Victoria, Australia, at the southern end of the Great Eastern Ranges corridor zone. We selected a group of vertebrate species representative of the region, developed alternative resistance surfaces based on species distribution models and expert opinion, respectively, and then used least-cost modelling for each species. We then combined the connectivity results for individual species to obtain an overall, multi-species connectivity map of the region, for each resistance surface. We sought to compare the extent of the differences that may arise when using two different types of resistance surface.

## Materials and methods

### Study area

**Fig 1:**
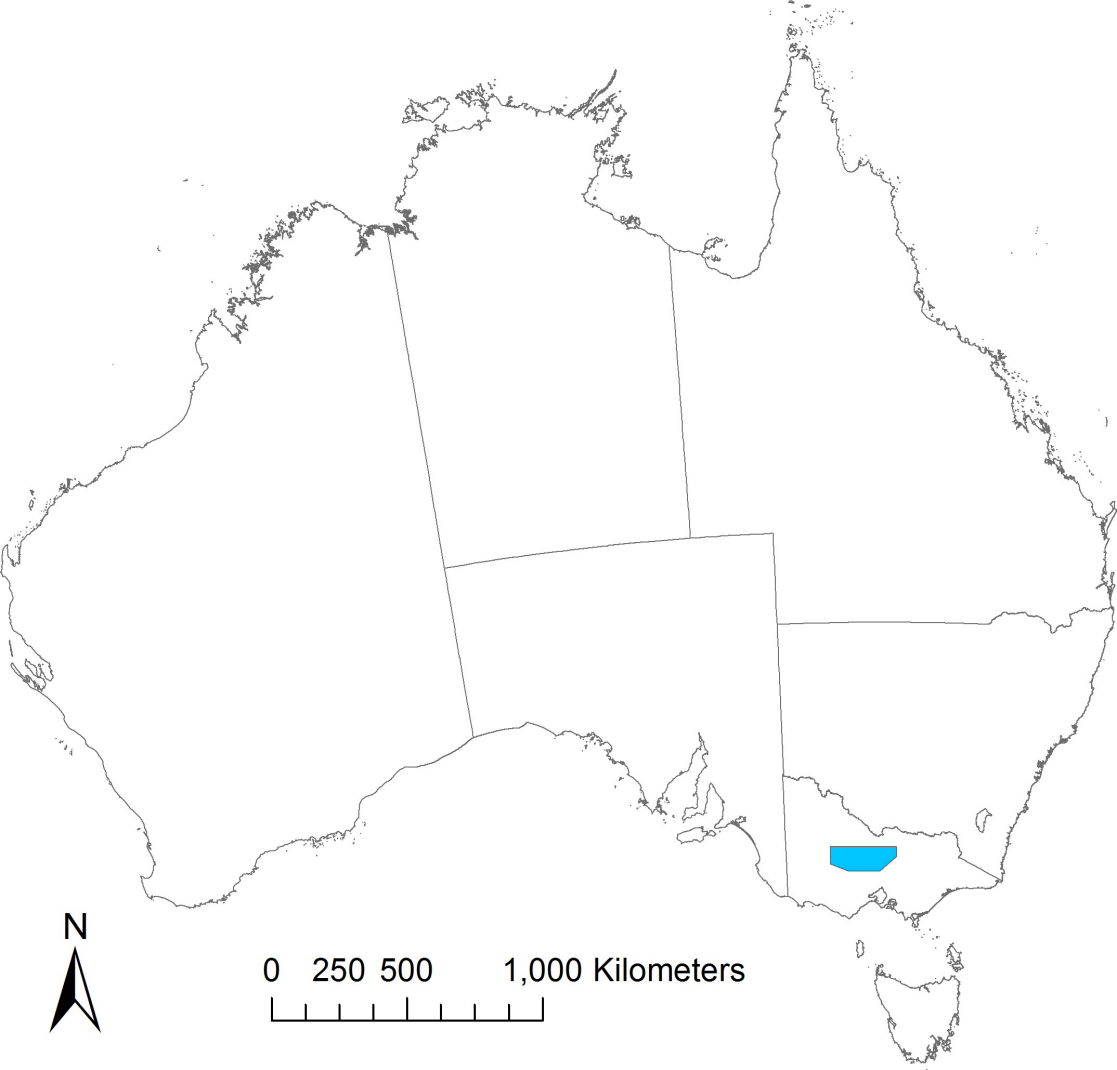
The study area. The area (in blue) is in north-central Victoria, Australia.

**Fig 2:**
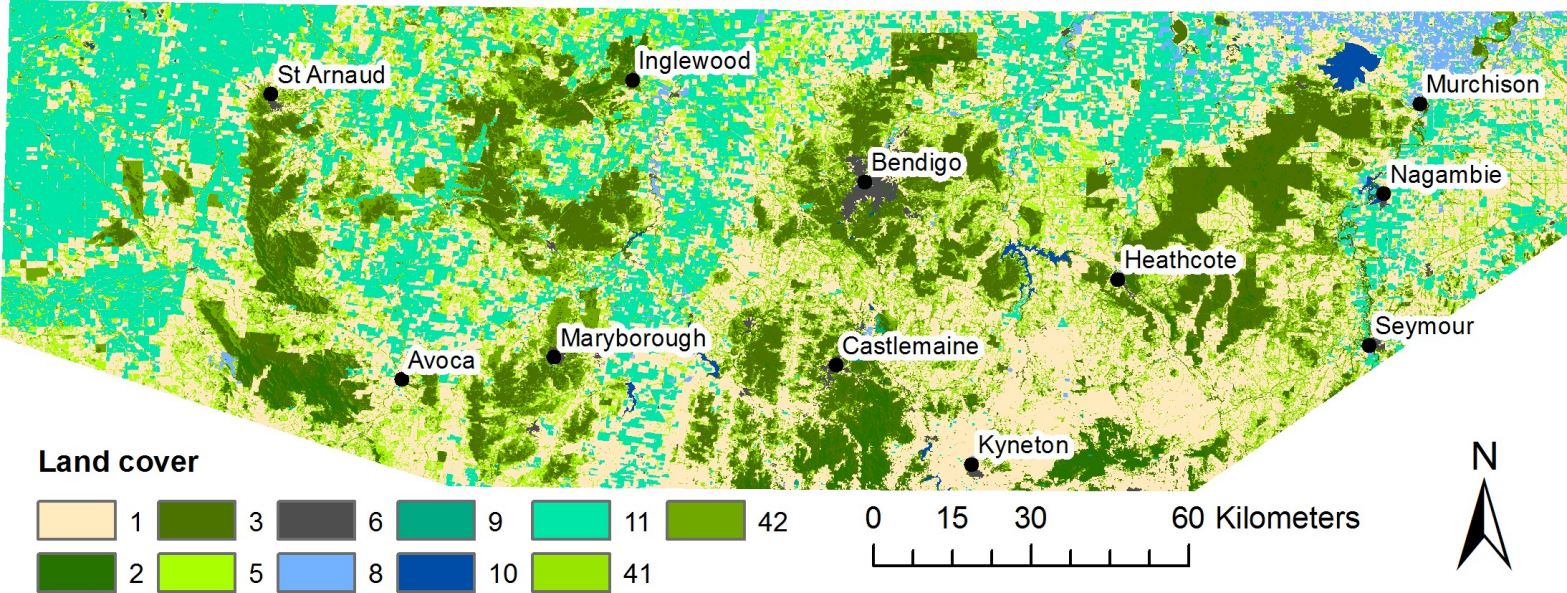
Land cover classes and major towns within the study area. Different land cover classes are shown by shading (see Table 1 for details). Continuous forest cover is shown in dark green.

The study area forms part of the box and ironbark region of north-central Victoria, Australia (17 969 km^2^, Fig 1) [51]. This area is representative of many regions in which fragmented blocks of native forest vegetation occur within a largely cleared agricultural environment, and support plant and animal communities with high conservation values (Fig 2). Additionally the region has been recognised as suitable for a broad ‘biolink zone’ [52, 53], it is within the Greater Eastern Ranges corridor zone supported by local community groups (e.g. Central Victorian Biolink, http://www.centralvicbiolinks.org.au/, accessed on 1 Oct 2014), and there is a good working knowledge of the biota of this region [51, 54].

**Table 1:** Resistance scores for each species for each land-cover class in the study area. Scores are the average of the values assigned by the experts. A higher score indicates greater resistance to the movement of a species through that land-cover class. Land cover classes include (1) Dryland pasture; (2) Dense forest cover; (3) Woodland and open forest; (4) Open woodland (which was split into 41 Open woodland–open woodland with numerous gaps and 42 Open woodland–where canopy vegetation is relatively continuous); (5) Temperate grassland and chenopod shrubland; (6) Urban–suburbs; (8) Irrigated crops, pasture and horticulture; (9) Plantation forestry; (10) Waterbodies and wetlands; and (11) Dry-land cropping.

Species	Land cover class										
	1	2	3	4(41)	4(42)	5	6	8	9	10	11
**Brush-tailed phascogale**	**86.0**	**1.4**	**1.0**	**47.6**	**15.2**	**71.2**	**94.0**	**94.0**	**59.2**	**96.8**	**90.0**
**Yellow-footed antechinus**	**91.8**	**1.4**	**1.0**	**51.6**	**14.2**	**77.0**	**93.0**	**95.0**	**60.2**	**96.0**	**94.0**
**Sugar glider**	**96.0**	**1.0**	**1.0**	**53.0**	**8.4**	**94.0**	**86.0**	**99.0**	**39.0**	**96.8**	**98.0**
**Buff-rumped thornbill**	**84.4**	**10.4**	**1.0**	**25.8**	**4.4**	**64.0**	**67.4**	**86.0**	**43.8**	**66.0**	**87.4**
**Grey shrike-thrush**	**77.8**	**1.8**	**1.0**	**13.2**	**4.0**	**53.6**	**45.0**	**59.8**	**18.0**	**51.4**	**83.6**
**Rufous whistler**	**58.2**	**1.8**	**1.0**	**15.4**	**4.6**	**47.0**	**34.8**	**65.4**	**9.8**	**49.0**	**62.0**
**White-throated treecreeper**	**85.6**	**1.6**	**1.0**	**37.2**	**10.0**	**78.0**	**64.4**	**91.0**	**28.2**	**73.0**	**89.2**
**Fuscous honeyeater**	**66.0**	**6.4**	**1.0**	**15.0**	**5.2**	**61.0**	**38.8**	**76.0**	**29.8**	**66.0**	**70.0**
**Wood gecko**	**90.0**	**16.6**	**1.8**	**49.0**	**11.2**	**52.0**	**86.0**	**98.0**	**69.0**	**98.0**	**97.0**
**Bougainville’s skink**	**88.0**	**9.6**	**1.0**	**37.2**	**3.6**	**38.0**	**86.0**	**90.0**	**62.0**	**98.0**	**93.0**
**Jacky lizard**	**95.8**	**8.2**	**1.0**	**44.2**	**8.7**	**65.8**	**90.8**	**94.0**	**65.8**	**97.5**	**97.5**
**Tree goanna**	**82.5**	**3.2**	**1.0**	**25.8**	**4.7**	**59.2**	**88.3**	**78.3**	**51.7**	**80.0**	**88.2**

### Study species

Our aim was to develop a general approach for identifying habitat networks for a region, and for the assemblage of species typical of north-central Victoria rather than for a particular flagship or umbrella species. We selected 12 species (see S1 Table for more information) that are relatively widespread and representative of the region, depend on wooded vegetation, represent different taxonomic groups, and have different levels of mobility. These included three species of mammals (brush-tailed phascogale *Phascogale tapoatafa*, sugar glider *Petaurus breviceps* and yellow-footed antechinus *Antechinus flavipes*), five species of birds (buff-rumped thornbill *Acanthiza reguloides*, fuscous honeyeater *Lichenostomus fuscus*, grey shrike-thrush *Colluricincla harmonica*, rufous whistler *Pachycephala rufiventris* and white-throated treecreeper *Cormobates leucophaeus*), and four reptile species (Bougainville’s skink *Lerista bougainvilliie*, jacky lizard *Amphibolurus muricatus*, tree goanna *Varanus varius* and wood gecko *Diplodactylus vittatus*).

### Defining core habitat patches

Our focus was primarily on species associated with forest or woodland habitats, rather than grasslands or wetlands that are best considered independently. Many possible approaches can be used to define habitat patches [1]. Initially, we attempted to define patches from a *species perspective*, rather than from mapped vegetation cover. This was based on the predicted species distribution from state-wide species distribution models (SDMs) [55]. However, this approach had important limitations. The patches produced tended to be too large and diffuse, because species distribution models tend to predict potential distributions rather than realized distributions.

We subsequently decided to map habitat patches based on land-cover classes, but to take into account the habitat preferences and spatial requirements of species. We used three land-cover classes relevant to the region and the study species, obtained from a state-wide land cover product with 10 classes [56]. These three classes were dense forest (Class 2), woodlands (Class 3) and open woodlands (Class 4, which was further split into two subclasses 41 and 42 based on continuity of canopy cover, see Table 1 for details). For each species, open woodlands (class 4) was processed further (because of its variation in habitat suitability): this involved intersecting the mapped species distribution model with locations having a predicted suitability above a threshold level (i.e. the top 40% of records were included) to define wooded patches where the species was likely to occur. Dense forest and woodland (Classes 2 and 3) and the processed open woodland (Class 4) were then combined. This resulted in too many patches (several thousands) for most species, which presented analytical challenges. Consideration of species-specific scales of habitat use was important: some patches were close to each other yet were likely to be functionally separated for some taxa; while for other taxa small gaps between the same patches may not disrupt the integrity of the habitat [1]. We used the GIS tool HCA [57] on the combined data to define ‘core’ habitat patches based on estimates of species home range size (see S1 Table) and by setting the minimum patch size as 20 ha. This approach produced habitat patches that were biologically meaningful at the scale of the study, and for which the number of patches (range: 157 to 978 across 12 species) was within the computational capacity available.

### Developing a resistance surface

A resistance surface is the primary input data for connectivity analyses, and represents the difficulty experienced by a species in moving across a landscape [24]. We used two approaches to develop resistance surfaces for each of the 12 species studied. The first approach was based on species distribution models [55]. We used the predicted habitat suitability for the species in each grid cell as a proxy of the likelihood that individuals will move through that cell (i.e. conductance), with values scaled from 0−100. The resistance value for each cell was then derived by subtracting the conductance value from 101 (i.e. scaled from 1−101).

The second approach for developing a resistance surface for each species used the land cover data as described above (Table 1), along with expert opinion on the ability of species to move through each cover class. Open woodland (Class 4) is a mixed class that contains woodland with different levels of tree density. To refine this class (to reflect its differing value to animal species), we used another state-wide dataset of tree cover developed from RapidEye satellite imagery [56]. The native resolution of this binary surface (tree / no tree) was 10 m × 10 m. This surface has a binary value of 1 if there are any trees in the cell, and 0 otherwise. This dataset was aggregated to a 75 m × 75 m resolution product, with summed values ranging from 0 to 64. Cells with values of 1–30 were reclassified as Open woodland–type A (Sub-class 41: open woodland with numerous gaps). Cells with values of 31–64 were classified as Open woodland–type B (Sub-class 42: canopy vegetation is relatively continuous). Cells with value 0 were grouped as Dryland Pasture (Class 1).

We sought assessments from 12 ecologists with extensive field experience with one or more of these faunal groups on the likely capacity of each species to move through each land-cover class (see Table 1). Movement capacity was scored on a range from 1 to 100, where 1 = uninhibited movement and 100 = totally inhibited movement. These estimates (Table 1) were combined by using a trimmed mean, omitting the highest and lowest values [23], and were used as the basis for producing an independent resistance surface for each species.

### Modelling connectivity

We used Linkage Mapper (LM) to model connectivity. Linkage Mapper is an ArcGIS extension tool designed to support regional analyses of wildlife habitat connectivity [58]. It uses GIS maps of core habitat patches and landscape resistances to identify and map linkages between core patches (http://www.corridordesign.org/designing_corridors/resources/gis_tools, accessed 1 Oct 2014). As animals move away from specific core areas, a cost-weighted distance analysis produces maps of the total movement resistance accumulated [58]. Linkage Mapper has been used in a range of large-scale connectivity analyses [14, 41, 59].

We used the cost-weighted and Euclidean network adjacency method and the nearest neighbour measurement unit in Euclidean distance, pruned the network to one connected nearest neighbour, and connected nearest pairs of core area “constellations”. Linkage Mapper requires that the width of corridors is specified for each focal species considered. Previous studies have posited a range of ideas for defining the scaling of appropriate corridor widths [25, 41, 60, 61]. Some researchers have suggested that a corridor should be as wide as, or even twice, the diameter of the species’ home range [61]. Given our focus on regional-scale connectivity, we adopted this approach of a broad width and set 2250 m as the corridor width for the brush-tailed phascogale and tree goanna, and 600 m for all the other species.

### Combining outputs from different species for a multi-species solution

We combined the outputs from different species for multi-species solutions. We first assigned a value of 1 to the cells through which a corridor passed and 0 to all the other cells. We then summed the results (transformed in this way) for all species when using the SDM-derived resistance surfaces, and likewise when using resistance surfaces based on expert opinion, respectively. We then used a ‘maximum’ neighbourhood analysis over a 1500 m × 1500 m zone. Focal cells took their final value as the number of species that had corridors intersecting the neighbourhood zone.

## Results

**Fig 3:**
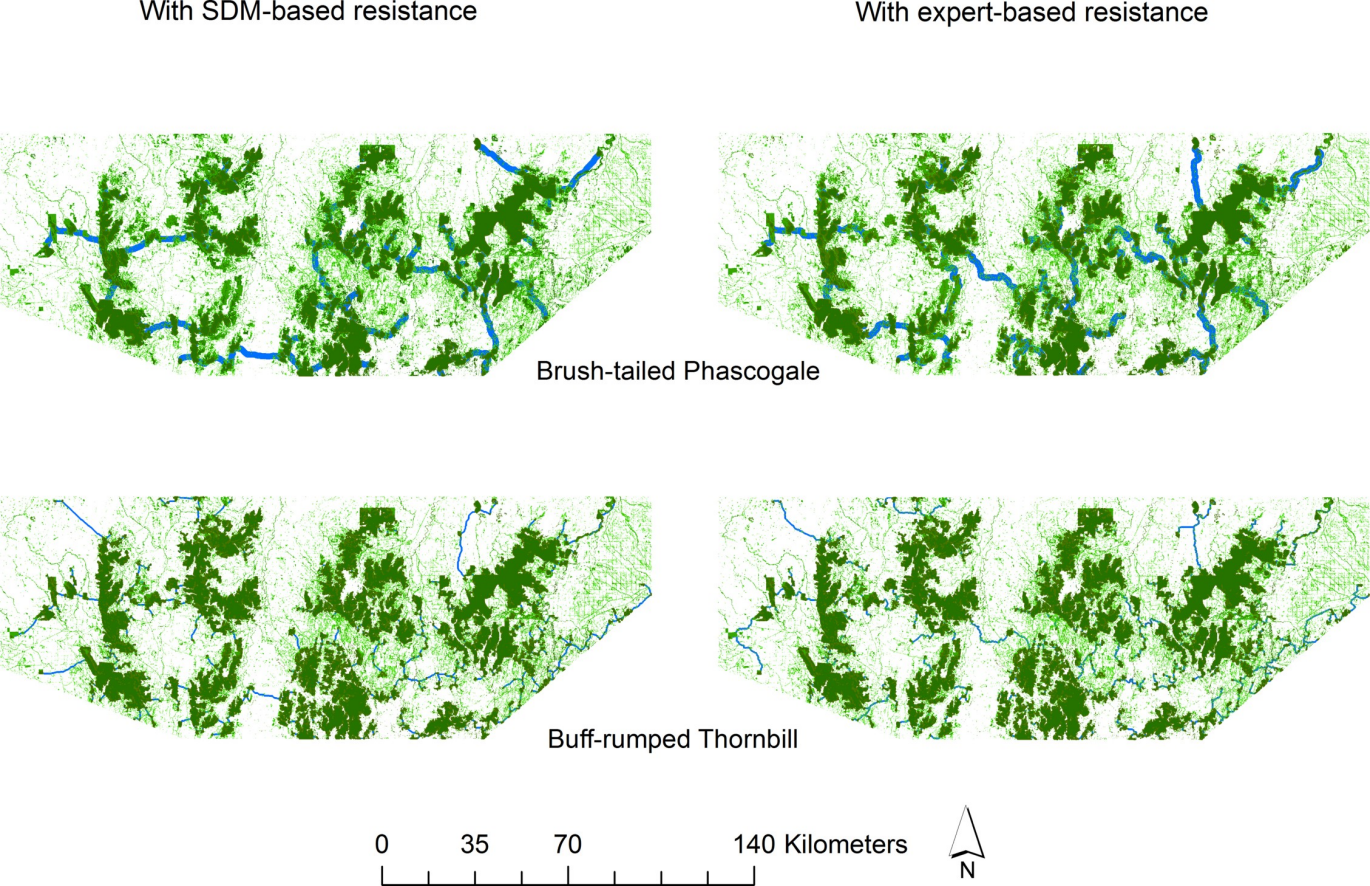
Least-cost paths identified for two species with two types of resistance surface. Left panel: from resistance surfaces based on species distribution models; Right panel: from resistance surfaces based on expert assigned scores; Top panel: for brush-tailed phascogale; Bottom panel: for buff-rumped thornbill. Habitat patches are shown in green, and least-cost paths are shown with blue lines.

Linkage Mapper analyses for each species, based either on resistance surfaces derived from SDMs or expert-assigned scores, produced generally similar results for most taxa when considering the whole study region at a coarse scale (Fig 3 and S1 and S2 Figs). At finer resolutions, differences existed. In more fragmented landscapes in the study area, linkages more closely followed strips of trees (e.g. along streams, roadsides) when using the expert-assigned resistance surfaces, whereas linkages arising from the SDM-based resistance surfaces displayed more diffuse patterns through the landscape.

**Fig 4:**
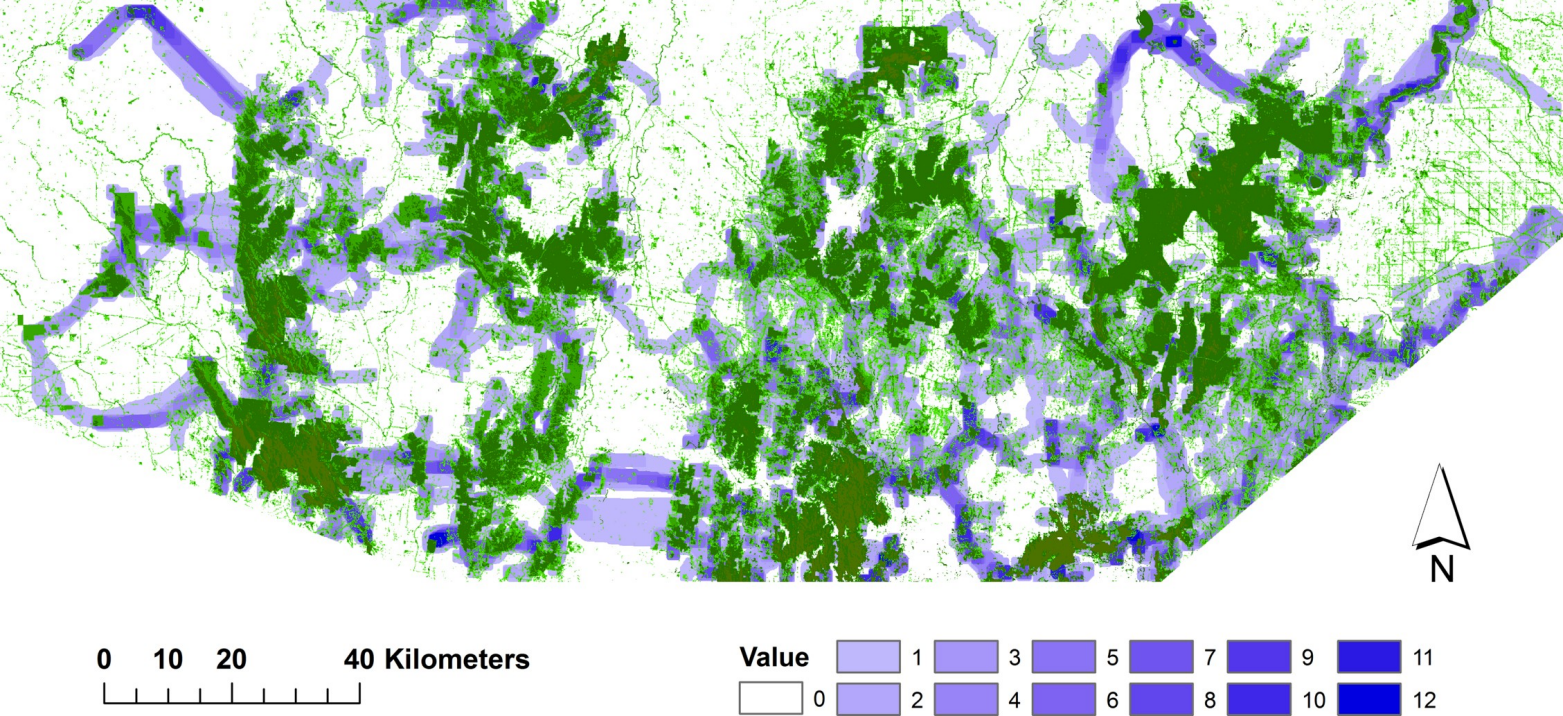
Composite regional connectivity map using resistance surfaces based on species distribution models. It is based on the combined results across all 12 study species. Favoured pathways are shown by darker blue shading, and existing wooded vegetation is in green.

**Fig 5:**
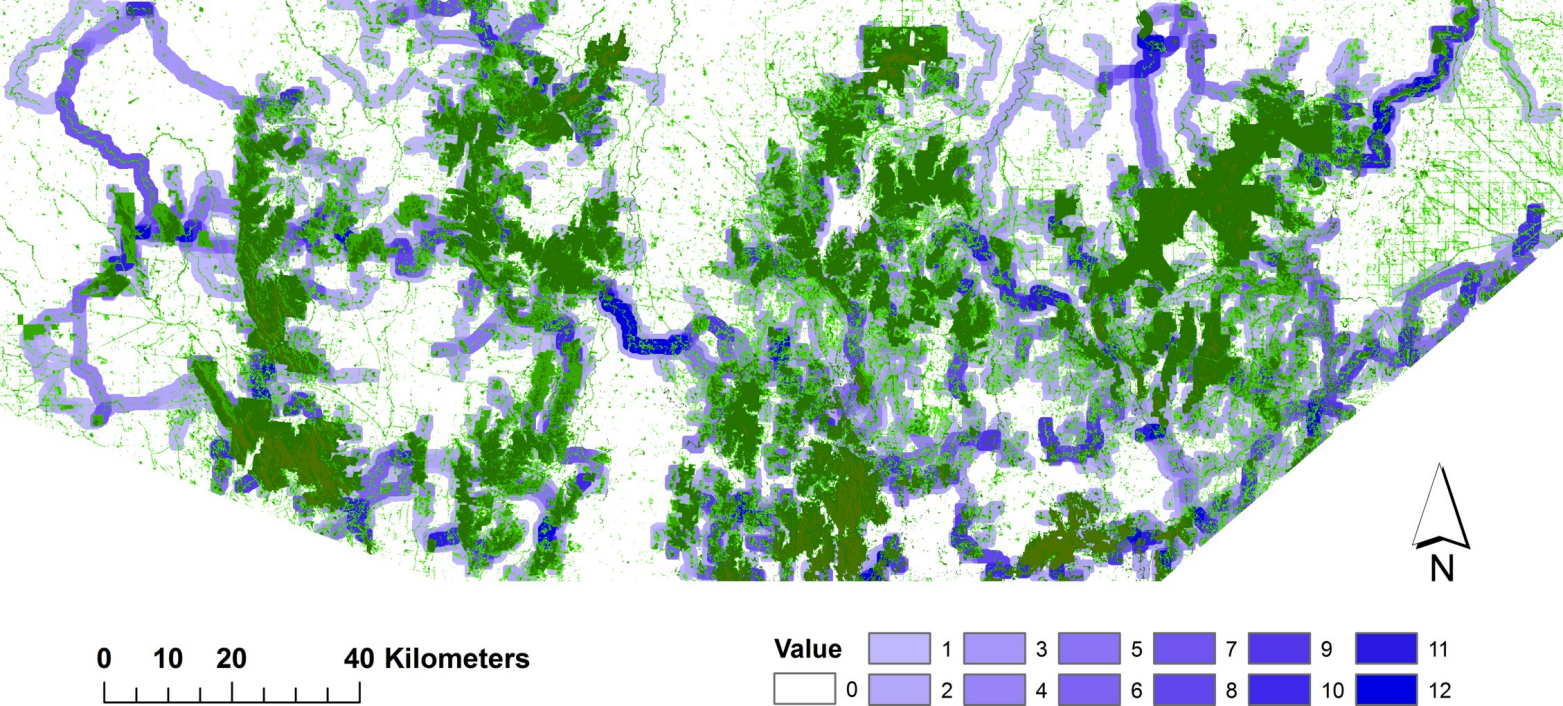
Composite regional connectivity map using resistance surfaces based on expert-assigned scores for land-cover classes. It is based on the combined results across all 12 study species. Favoured pathways are shown by darker blue shading, and existing wooded vegetation is in green.

The results from combining the movement paths of all species highlight the key linkage pathways that will benefit multiple species (Figs 4 and 5). While there was a general similarity between the maps obtained by using different resistance surfaces (e.g. in the southwest and southeast), differences relate to the greater propensity for those derived from the resistance surface based on expert-assigned scores for land cover classes, to follow existing vegetation. A notable example occurs in the centre of the study region (Fig 5) where a single favoured path was identified by Linkage Mapper to link the eastern and western parts of the region. This pathway followed strips and patches of remnant wooded vegetation along a river system (the Loddon River) (Fig 5). In contrast, the use of the SDM-based resistance surface (Fig 4) identified several (less-favoured) pathways between eastern and western parts of the region, mostly in the south centre, corresponding with the shortest gaps between large forested areas but requiring crossing of tracts of cleared farmland.

## Discussion

### Connectivity modelling for multiple species

We used one of the most popular methods − a least-cost path modelling method implemented with Linkage Mapper, to carry out an assessment of connectivity for multiple species of vertebrates using two alternative resistance surfaces.

An important feature of our approach was to model connectivity for multiple species of vertebrates and to combine the outputs of these different models at the regional scale. A growing interest in modelling multi-species connectivity reflects a view that conservation measures to maintain or restore connectivity will be of greatest value if they can create a robust network of habitats for sets of species that have similar requirements [41, 44] or for the overall assemblage of fauna [40]. Thus, the focus is less on enhancing movement per se for large or wide-ranging species [22, 32] but on creating a connected network of habitats for long-term conservation.

A key challenge for multispecies connectivity is to determine the most suitable form and location for linkages, given that different species have different habitat requirements and scales of movement. One approach is to identify connecting pathways that have greatest landscape integrity, avoiding barriers or highly modified land cover [1, 9, 40, 59], rather than modelling the actual requirements of particular species. However, like others [34, 43, 46], we chose to model connectivity for multiple species on an individual basis and then to combine these results. Our approach was to overlay the pathways derived for individual species, giving weight to locations that benefit more species. This allows choices to be made among alternatives, rather than being a prescriptive map. On-ground implementation of corridors must take into account a number of factors, such as land tenure, existing vegetation and the conservation values of different fragmented systems (see below).

### Resistance surfaces

We used two approaches to construct resistance surfaces: one based upon species distribution models for the focal taxa, and the other based on expert opinion of the ability of these taxa to move through different land-cover classes. The primary difference was that, with the latter, the pathways identified more closely followed existing strips and patches of vegetation, typically remnant eucalypt woodland along streams and roadsides, 20–80 m in width. This was much less apparent for SDM-based resistance surfaces: the species distribution models did not always recognise such strips as suitable habitat because vegetation cover was not included in building the models [55].

Recognising such existing vegetation in land cover mapping, and planning and creating linkages that incorporate it has a number of benefits. First, it is more efficient to build on the natural infrastructure already present, most of which is on public land and therefore more amenable to such planning than private land. Second, much of the streamside and roadside vegetation already occurs as connected networks in the landscape. Third, these remnant linear strips are known to provide habitat for a range of bird, mammal and reptile species [62–65]. Last, there are multiple benefits from retaining and expanding such strips: streamside vegetation, for example, also enhances aquatic environments and reduces stream bank erosion.

In most situations, empirical data on movement pathways, gene flow or genetic distances are unavailable, and so expert opinion becomes the only realistic option on which to base a resistance model [23, 24]. Here, the combination of mapped land cover classes combined with expert assessment of the likely use of each class, is a useful and realistic approach. Nevertheless, uncertainty remains with the use of expert opinion, and so further enhancement can be expected where quantitative data are available on the relative suitability and capacity for movement by species in different vegetation classes, and on gap-crossing abilities [66].

### Application to regional planning

Connectivity models, such as those employed here, are a first step in developing regional-scale plans for enhancing connectivity. Other considerations also come into play in determining where and when practical conservation actions will be undertaken, the type of actions to be implemented, and who will carry them out.

The modelling approaches used here identify pathways between *all* core habitat patches included in the analysis, whether large or small, close or isolated from other blocks. The priority given to each proposed corridor in regional planning will vary, depending on the ecological and conservation values of the core habitats they connect and the feasibility and cost of implementing actions. Some pathways may be assigned high priority on ecological grounds because they connect large core habitats which have high conservation value to a regional network. Others may have high priority because they have both high conservation value and a high feasibility of being implemented.

Major regional corridors of the scale mapped here (e.g. 0.6–2.2 km wide, a few to several kms in length) require that large tracts of land are managed for conservation purposes. A key limitation is land tenure, particularly when much of the land identified is privately owned. This requires either resources for land acquisition and management, voluntary efforts by the landholder, or the provision of incentives to manage land for conservation purposes. In this region, many of the important pathways (Fig 5) follow streams and rivers, which have associated public land buffers on either side. This offers an opportunity to build the framework of a network and to consolidate over time. There are multiple benefits, in addition to wildlife conservation and connectivity, from wide belts of riparian vegetation [67].

Finally, the presence and quality of existing vegetation will influence the regional priority given to protection and restoration. The presence of existing small patches and strips of vegetation that can be integrated into a regional corridor network offer the opportunity for community action to build on these incrementally through protection, restoration and expansion over time. A longer-term perspective for such regional planning and implementation of regional connectivity is essential.

## Supporting information

S1 Table Summary of information on estimated home range and gap-crossing ability for the selected study species. (DOCX)

S1 Fig Identified linkages with two types of resistance surfaces for five species. The resistance surfaces were based on species distribution models and expert-assigned scores. The five species are Bougainville’s skink, fuscous honeyeater, grey shrike-thrush, jacky lizard and rufous whistler. Habitat patches are shown in green, and least-cost paths are shown with blue lines.
(TIF)

S2 Fig Identified linkages with two types of resistance surfaces for five species. The resistance surfaces were based on species distribution models and expert-assigned scores. The five species are sugar glider, tree goanna, wood gecko, white-throated treecreeper and yellow-footed antechinus. Habitat patches are shown in green, and least-cost paths are shown with blue lines.
(TIF)
