# Supplementary material for: Identifying wildlife corridors for the restoration of regional habitat connectivity: A multispecies approach and comparison of resistance surfaces
Source: PLoS One. 2018 Nov 7;13(11):e0206071. doi: 10.1371/journal.pone.0206071 (PMC6221308; doi:10.1371/journal.pone.0206071)
Supplement: S1 Table — (DOCX) [file pone.0206071.s001.docx]

**S1 Table**. **Summary of information on estimated home range and gap-crossing ability for the selected study species.**

| **Species** | Home range (ha) | Gap crossing (conservative, m) | Number of patches* |
| --- | --- | --- | --- |
| *Mammals* |  |  |  |
| Brush-tailed phascogale | 100 | 250 | 155 |
| Yellow-footed antechinus | 2 | 250 | 470 |
| Sugar glider | 4 | 250 | 506 |
| *Birds* |  |  |  |
| Buff-rumped thornbill | 5 | 100 | 561 |
| Grey shrike-thrush | 6 | 100 | 554 |
| Rufous whistler | 4 | 100 | 978 |
| White-throated treecreeper | 4 | 100 | 511 |
| Fuscous honeyeater | 4 | 100 | 618 |
| *Reptiles* |  |  |  |
| Wood gecko | <1 | 100 | 351 |
| Bougainville’s skink | <1 | 100 | 513 |
| Jacky lizard | 1-3 | 100 | 470 |
| Tree goanna | 100 | 250 | 158 |

Note: *Minimum patch size is 20 ha.
